# A Prospective Randomized Study Comparing Mini-surgical Percutaneous Dilatational Tracheostomy With Surgical and Classical Percutaneous Tracheostomy: A New Method Beyond Contraindications: Erratum

**DOI:** 10.1097/01.md.0000479439.98820.8e

**Published:** 2016-01-08

In the article “A Prospective Randomized Study Comparing Mini-surgical Percutaneous Dilatational Tracheostomy With Surgical and Classical Percutaneous Tracheostomy: A New Method Beyond Contraindications”,^1^ which appeared in Volume 94, Issue 47 of *Medicine*, the links for 2 supplementary videos were not provided in the “Tracheostomy Procedure” section. The article has since been corrected online.
